# Advance in identified targets of berberine

**DOI:** 10.3389/fphar.2025.1500511

**Published:** 2025-01-29

**Authors:** Penghai Sun, Ziyuan Wang, Yinchao Ma, Yuan Liu, Yintong Xue, Yan Li, Xiang Gao, Yuedan Wang, Ming Chu

**Affiliations:** ^1^ Department of Immunology, School of Basic Medical Sciences, Peking University, NHC Key Laboratory of Medical Immunology (Peking University), Beijing, China; ^2^ Beijing Life Science Academy, Beijing, China; ^3^ Department of Gastroenterology, Peking University Third Hospital, Beijing, China; ^4^ Beijing Key Laboratory for Helicobacter Pylori Infection and Upper Gastrointestinal Diseases, Peking University Third Hospital, Beijing, China

**Keywords:** berberine, ftsZ, QacR, BmrR, PLA2, ramR, Nek7, met

## Abstract

Berberine is an isoquinoline alkaloid, which has demonstrated significant therapeutic potential in the treatment of various diseases, including tumors, acute and chronic infections, autoimmune disorders, and diabetes. Studies have demonstrated that berberine exhibits polypharmacological effects, including antibacterial, anti-inflammatory, antioxidant, and hypoglycemic activities. To further elucidate the multifaceted pharmacological mechanisms of berberine, we reviewed 7 targets of berberine identified through co-crystal structure analysis, including filamentous temperature-sensitive protein Z (FtsZ), QacR, BmrR, phospholipase A2 (PLA2), RamR, NIMA-related kinase 7 (NEK7), and mesenchymal-epithelial transition (MET). Through target fishing, molecular docking, and surface plasmon resonance (SPR) analyses, combined with cellular and molecular experiments, we further identified 6 targets of berberine. These findings provide a comprehensive summary of berberine’s direct molecular targets, offering a theoretical foundation for further exploration of its diverse pharmacological activities.

## 1 Introduction

Berberine, an isoquinoline alkaloid found in *Berberidaceae*, *Ranunculaceae*, and *Papaveraceae*, was initially utilized for the treatment of diarrhea (Singh and Mahajan, 2013). Notably, accumulating evidence has demonstrated that berberine plays a significant role in managing diverse conditions, including diabetes, hyperlipidemia, gastrointestinal infections, cancer, and Alzheimer’s disease (Wang et al., 2022; Shen et al., 2020; Xiong et al., 2022; Sun C. et al., 2024; Goel, 2023). These therapeutic effects are attributed to its polypharmacological effects, including antimicrobial, anti-inflammatory, antioxidant, and hypoglycemic activities (Ehteshamfar et al., 2020; Fatahian et al., 2020; Chu et al., 2014; Mombeini et al., 2022; Ilyas et al., 2020). Mechanistically, berberine primarily regulates key signaling pathways, including nuclear factor-κB (NF-κB), janus kinases (JAK)/Signal transducer and activator of transcriptions (STAT), mitogen-activated protein kinases (MAPK), adenosine 5′-monophosphate (AMP)-activated protein kinase (AMPK)/mammalian target of rapamycin (mTOR), phosphatidylinositol 3-kinase (PI3K)/AKT, and other signaling pathways, to exert these diverse pharmacological effects (Chen et al., 2017; Haftcheshmeh et al., 2022; Sun A. et al., 2024). Focusing on identified targets of berberine, we chose seven targets that met the inclusion criteria based on co-crystal structure analyses. These targets include QacR, BmrR, the d (CGTACG)2 DNA sequence, PLA2, RamR, NEK7, and MET [(Schumacher et al., 2001; Newberry et al., 2008; Yamasaki et al., 2013; Chandra et al., 2012; Ferraroni et al., 2011; Song et al., 2022; Chen et al., 2022; Zeng et al., 2021)]. In 2008, Prerna N. Domadia et al. identified that berberine targets FtsZ by binding to its hydrophobic pocket, thereby disrupting the formation of the Z-ring (Domadia et al., 2008). Kate M. Peters et al. proposed that the multiple drug-binding pockets of QacR exhibit multifunctionality, allowing interactions with various cationic drugs, including berberine, through multiple binding modes in 2008 [(Schumacher et al., 2001; Newberry et al., 2008; Yamasaki et al., 2013; Chandra et al., 2012; Ferraroni et al., 2011; Song et al., 2022; Chen et al., 2022; Zeng et al., 2021)]. Similarly, Newberry et al. identified berberine as a natural activator of BmrR, offering critical insights into its interaction with BmrR and its role in regulating bacterial resistance (Newberry et al., 2008). In 2011, Ferraroni et al. first reported the crystal structure of berberine in complex with the d (CGTACG)2 DNA sequence (Ferraroni et al., 2011). Subsequently, in 2012, D. Naveen et al. demonstrated through SPR analysis that berberine binds to phospholipase A2(PLA2) in a concentration-dependent manner (Chandra et al., 2012). In 2013, Yamasaki et al. resolved the crystal structure of the RamR-berberine complex, highlighting its relevance to bacterial resistance (Yamasaki et al., 2013). In 2020, Zeng et al. showed that berberine directly binds to NEK7, inhibiting the NEK7- nucleotide-binding oligomerization domain-like receptor protein 3 (NLRP3) interaction and thereby exerting anti-inflammatory effects (Zeng et al., 2021). Furthermore, in 2022, Chen et al. found that berberine acts as a direct MET inhibitor, playing a pivotal role in the treatment of non-small cell lung cancer (NSCLC) (Chen et al., 2022).

In 2018, we proposed the Drug-Target Space (DTS) model, establishing the foundation for AI-based drug-target screening (Chu et al., 2018). Building on this framework, we identified candidate targets of berberine. Using SPR, molecular docking, along with cellular and animal experiments, we confirmed that beta-site amyloid precursor protein cleaving enzyme (BACE1) and amyloid beta1-42 (Aβ1-42) are direct targets of berberine, elucidating its pharmacological basis in the treatment of Alzheimer’s disease. Subsequently, we identified additional berberine targets, including myeloid differentiation 2 (MD-2), phenol-soluble modulins alpha 2(PSMα2), transforming growth factor-beta receptor 1 (TGFBR1), and Janus kinase 2 (JAK2). These findings have unveiled novel mechanisms underlying berberine’s polypharmacological actions, particularly in the context of its antimicrobial and anti-inflammatory effects, as well as its therapeutic potential in Alzheimer’s disease, pancreatic cancer, lung metastasis, and myasthenia gravis (Chu et al., 2014; Song et al., 2022; Chu et al., 2018; Chu et al., 2016; Tian et al., 2022).

## 2 Ftsz

FtsZ (filamentous temperature-sensitive protein Z) is a key protein involved in cell division in bacteria. In 2008, Prerna N. Domadia and colleagues demonstrated that berberine directly targets *Escherichia coli* FtsZ, inhibiting the dynamics of Z-ring assembly and disrupting the process of cell division in bacteria. In their study, berberine exhibited a high binding affinity to FtsZ, with a dissociation constant (KD) of 0.023 μM at an FtsZ concentration of 10 μM. Berberine was found to interact with hydrophobic residues near the GTP-binding pocket of FtsZ, including Pro134, Phe135, Phe182, Leu189, Ile163, and Pro164 [(Schumacher et al., 2001; Newberry et al., 2008; Yamasaki et al., 2013; Chandra et al., 2012; Ferraroni et al., 2011; Song et al., 2022; Chen et al., 2022; Zeng et al., 2021)]. Notably, in 2023, Angela Di Somma et al. synthesized a series of berberine derivatives with enhanced antibacterial activity by targeting FtsZ, underscoring the potential of FtsZ-targeting compounds for the development of more effective antimicrobial agents (Di Somma et al., 2023).

## 3 QacR

**FIGURE 1:**
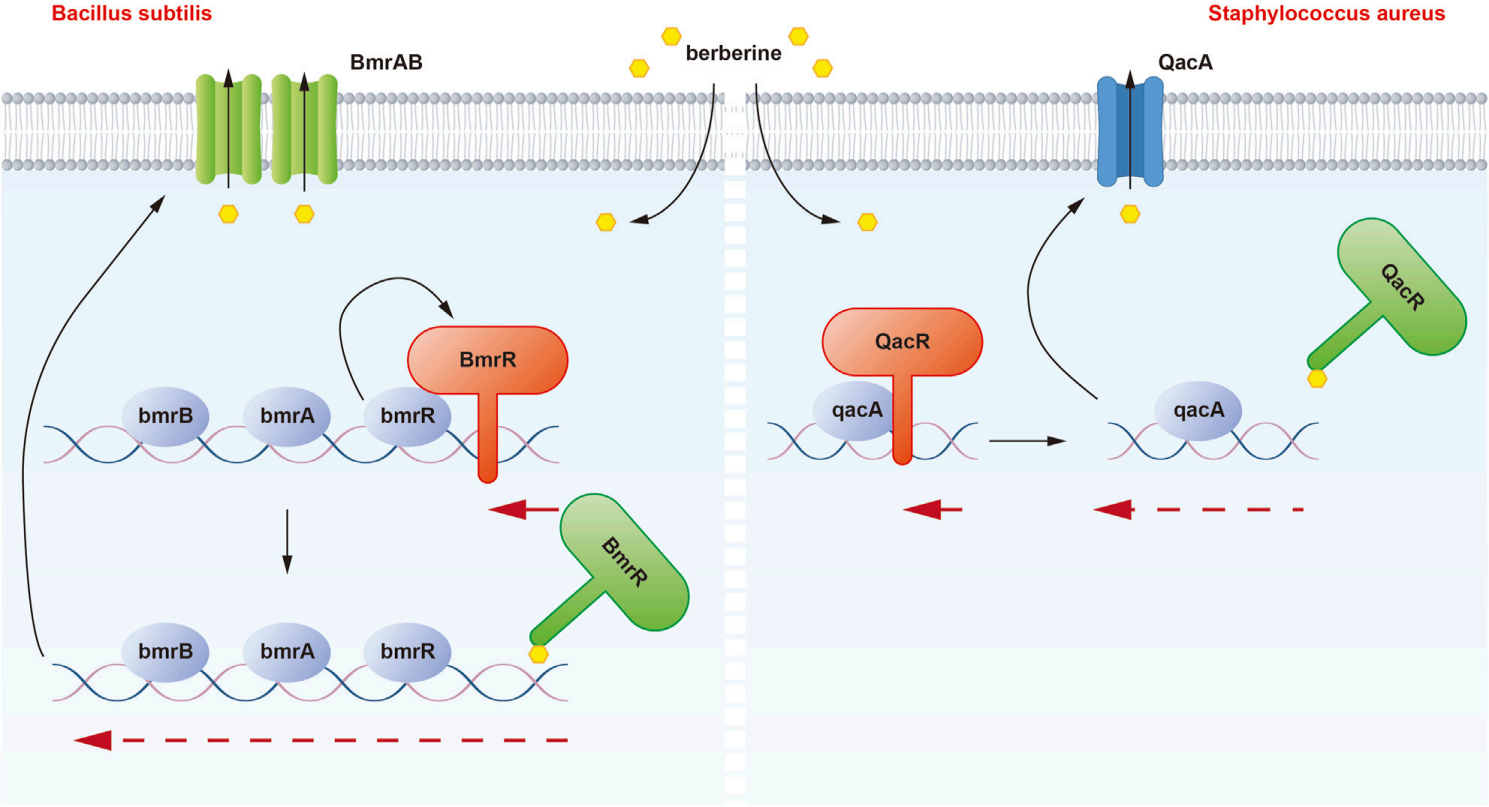
Illustration of the binding mechanism of berberine with QacR in *Staphylococcus aureus* and BmrR in *Bacillus subtilis*. In the presence of berberine, the inhibitory effect of QacR on qacA expression is relieved. Under normal conditions, BmrR binds to the bmr box and represses transcription of the BmrRAB operon, which is disrupted when berberine binds to BmrR. These modifications result in the transcription of BmrAB and QacA transporters to pump out berberine.

The binding interaction between berberine and QacR was first demonstrated. QacR is a protein associated with multidrug resistance and is found in *Staphylococcus aureus* (Figure 1). The qacA gene encodes QacA, a multidrug efflux protein, which can expel various toxic compounds from bacterial cells, leading to bacterial resistance. QacR regulates the expression of multidrug resistance by inhibiting the transcription of the qacA multidrug transporter gene (Forman et al., 2016). In 2008, Peters and colleagues proposed that QacR primarily interacts with berberine through E57 and E58 glutamic acid residues. Moreover, different cationic drugs binding to the QacR pocket can adopt distinct positions to neutralize charges. When cationic lipophilic drugs bind to QacR, the protein undergoes a conformational change, forming a multidrug-binding pocket (Schumacher et al., 2001). Within this pocket, glutamic acid residues and aromatic residues mediate drug interactions. QacR can interact with various cationic drugs through multiple mechanisms (Peters et al., 2008).

## 4 BmrR

Berberine can enter the drug-binding pocket of BmrR and bind to it. Berberine may play a significant role in the regulation of bacterial resistance by binding to and activating the BmrR protein (Figure 1). BmrR belongs to the mercuric -responsive transcriptional regulator (MerR) family of multidrug-binding transcription factors and influences the function of the multidrug efflux pump Bmr by regulating the expression of the bmr gene, thereby affecting bacterial resistance (Wade, 2010). In 2008, Newberry et al. discovered that berberine could form a complex with the BmrR protein, and its binding site was similar to that of drugs like R6G. Structural studies of the BmrR-Ber-DNA complex revealed that berberine’s orientation in the drug-binding pocket is such that its acridine system is wedged between Trp61 and Tyr93, while the 1,3-dioxo-6a-azaniumylindole moiety stacks with Tyr123. Additionally, the positive charge center of berberine is situated on the N1 nitrogen of the Be ring and is surrounded by the side chains of Glu57 and Glu58. Berberine forms a hydrogen bond with P144 through its carbonyl oxygen, further contributing to complex formation.

## 5 DNA

Berberine’s antimicrobial, anti-inflammatory, antioxidant, and anticancer activities have been extensively reported, primarily attributed to its ability to form complexes with DNA [(Ferraroni et al., 2011; Vlavcheski et al., 2022; Devarajan et al., 2021)]. In 2003, Mazzini and colleagues investigated the interactions between berberine and double-stranded oligonucleotides, including d (AAGAATTCTT)2, d (GCGATCGC)2, d (CGTATACG)2, d (CGTACG)2, 5′-d (ACC​TTT​TTG​ATG​T)-3′/5 (ACATCAAAAAGGT)-3′, as well as single-stranded 5′-d (ACATCAAAAAGGT)-3′, using 1H, 31P NMR, and UV spectroscopic techniques. They found that berberine tended to bind to DNA sequences rich in AT base pairs (Mazzini et al., 2003). In 2011, Ferraroni and colleagues reported the crystal structure of berberine with the d (CGTACG)2 DNA sequence. In 2021, Wickhorst and others discovered that berberine derivatives substituted with 9- and 12-dimethylaminophenyl groups exhibited strong binding affinity to quadruplex DNA. Furthermore, these derivatives exhibited differential binding modes and pH-dependent effects on nucleic acids. Unlike the original berberine, which exhibited enhanced DNA binding at neutral conditions, these derivatives showed stronger binding at pH 5 [(Schumacher et al., 2001; Newberry et al., 2008; Yamasaki et al., 2013; Chandra et al., 2012; Ferraroni et al., 2011; Song et al., 2022; Chen et al., 2022; Zeng et al., 2021)].

## 6 PLA2

**FIGURE 2:**
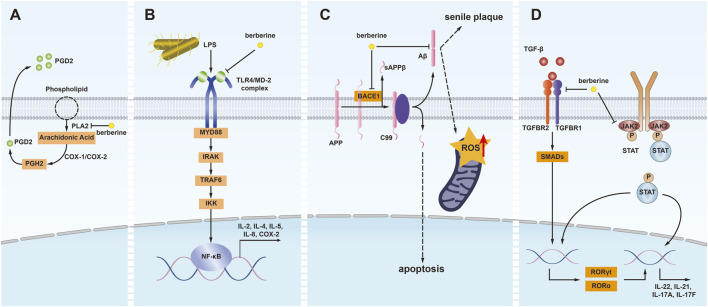
Illustrating of the binding mechanisms of berberine with PLA2, MD2, BACE1, Aβ1-42, TGFBR1, and JAK2 in human cells. **(A)** Phospholipase A2 (PLA2) converts phospholipids into arachidonic acid, which is then transformed into prostaglandin H2 (PGH2) by cyclooxygenase-1/2 (COX-1/2) and subsequently into prostaglandin D2 (PGD2) by hematopoietic prostaglandin D synthase (HPGDS). Berberine exhibits anti-inflammatory effects by inhibiting PLA2, thereby reducing arachidonic acid production. **(B)** Lipopolysaccharides (LPS) initiate inflammatory responses via the Toll-like receptor 4 (TLR4)/MD-2 signaling pathway. Berberine disrupts this pathway by binding to MD-2, mitigating the inflammatory response. **(C)** In the amyloidogenic pathway, amyloid precursor protein is cleaved by beta-secretase 1 (BACE1) to generate amyloid-beta (Aβ) peptides. The accumulation and aggregation of Aβ peptides lead to neurotoxic amyloid plaques, inducing senile plaques, apoptosis, and increased reactive oxygen species (ROS) within mitochondria. Berberine inhibits BACE1 and Aβ, exerting anti-aging and antioxidant effects. **(D)** Transforming growth factor-beta (TGF-β) binds to heteromeric receptor complexes composed of TGF-β receptor 1 (TGFBR1) and TGF-β receptor 2 (TGFBR2), triggering signal transduction. This leads to the phosphorylation of SMAD family members, which then migrate to the nucleus to regulate gene expression. Berberine inhibits the binding of TGF-β to its receptor complex, reducing the expression of inflammatory genes, including RORγt, RORα, and IL-22. Additionally, berberine inhibits the phosphorylation of STAT proteins by inhibiting Janus kinase 2 (JAK2), further exerting anti-inflammatory effects.

In 2012, Chandra et al. conducted surface plasmon resonance analysis and found that berberine bound to Phospholipase A2 (PLA2) in a concentration-dependent manner, with a measured equilibrium dissociation constant (KD) of 5.55 × 10-8M. Additionally, through molecular docking experiments, Chandra et al. identified the active site residues on ppPLA2 that came into contact with berberine. The most crucial residues involved in this interaction included G32, R53, D49, Y69, Y52, H48, G33, S34, and P68. Furthermore, when berberine was biotransformed by Rhizopus oryzae, the resulting hydroxylated derivatives of berberine exhibited stronger binding affinity and inhibitory effects on PLA2. This altered the way berberine interacted with the active site of PLA2, making it more favorable for berberine to bind to the protein’s active site (Chandra et al., 2012). PLA2 belongs to the class of lipolytic enzymes and hydrolyzes the ester bond at the sn-2 position of phosphatidylcholine. During hydrolysis, PLA2 releases fatty acids such as arachidonic acid (AA), participating in processes that alter cell membrane structure and playing a crucial role in inflammation, cell signal transduction, and carcinogenesis (Khan and Ilies, 2023; Cathcart et al., 2011; Peng et al., 2021) (Figure 2A).

## 7 RamR

In 2013, Yamasaki et al. reported the crystal structure of the complex formed between RamR and berberine. They determined that the KD value for the binding of berberine to RamR was 17.9 ± 0.03 μM using surface plasmon resonance experiments. After bacterial cells were treated with berberine, the promoter activity of RamR was enhanced. Similar to other drugs, berberine’s Phe155 and RamR’s Phe155 were found to be parallel, indicating that they interacted with RamR through π–π stacking interactions (Yamasaki et al., 2013). In a manner analogous to the mechanisms observed with QacR and BmrR, RamR is a transcriptional repressor of the RamA protein gene, which regulates the expression of the multidrug efflux system genes acrAB-tolC and is an important factor in multidrug resistance. When berberine is used in antibacterial therapy, it activates RamR, resulting in the upregulation of the acrAB-tolC system, which enhances bacterial resistance. In 2022, Jyoti Mehta and colleagues discovered that methanol extracts of Diospyros lotus (a medicinal plant) could inhibit the AcrAB-TolC efflux pump activity in *Salmonella enterica* serovar Typhimurium, leading to a 2- to 4-fold increase in the antibacterial potency of berberine (Mehta et al., 2022).

## 8 NEK7

In 2020, Zeng et al. reported that berberine directly bind to and target NIMA-related kinase 7 (NEK7) protein to block NEK7-NLRP3 interaction, achieving anti-inflammatory efficacy in a NEK7-dependent manner with an IC50 of 4.2 μM [(Schumacher et al., 2001; Newberry et al., 2008; Yamasaki et al., 2013; Chandra et al., 2012; Ferraroni et al., 2011; Song et al., 2022; Chen et al., 2022; Zeng et al., 2021)]. Researchers have conducted numerous significant studies on the mechanisms by which NEK7 regulates the NLRP3 inflammasome signaling pathway. These pathways include potassium efflux, ROS signaling, lysosomal destabilization, and NF-κB signaling (He et al., 2016; Sharif et al., 2019; Gross et al., 2016; Chen et al., 2019; Liu et al., 2020). NEK7 is considered a potential therapeutic target for NLRP3-related diseases, and inhibitors targeting NEK7, such as berberine, may suppress inflammatory responses by modulating NLRP3 [(Zeng et al., 2021; Jin et al., 2023)].

## 9 MET

The inhibitory effect of berberine on the MET gene has been demonstrated. MET is a proto-oncogene that encodes the transmembrane receptor for hepatocyte growth factor (HGF). MET exhibits tyrosine kinase (TK) activity, and the MET tyrosine kinase is the only known high-affinity receptor for HGF. The HGF/MET signaling pathway is well characterized and recognized for its essential role in carcinogenesis and tumour progression (Gowda et al., 2024; Kumar et al., 2024). Studies have shown that MET amplification is among the most common mediators of TKI resistance (Peng et al., 2019; Hartmaier et al., 2023). Therefore, the study of MET inhibitors holds significant importance for the treatment of diseases such as non-small cell lung cancer (Yun et al., 2020). In 2022, Chen et al. found that berberine can act as a naturally-existing MET inhibitor to synergize with osimertinib in overcoming osimertinib acquired resistance caused by MET amplification (Chen et al., 2022). Furthermore, they showed that berberine inhibits MET activity in a dose-dependent manner, with an IC50 of 19.64 μM. Further research on berberine derivatives plays a crucial role in the future development of more optimized MET inhibitors.

## 10 PSMα2

In 2016, we found that berberine inhibited the formation of amyloid-like fibers in *S. aureus*, including PSMs. Further molecular dynamics simulations revealed that berberine could bind to the phenyl ring of Phe19 in PSMα2 [(Schumacher et al., 2001; Newberry et al., 2008; Yamasaki et al., 2013; Chandra et al., 2012; Ferraroni et al., 2011; Song et al., 2022; Chen et al., 2022; Zeng et al., 2021)]. Phenol-soluble modulins (PSMs) are important virulence factors in *S. aureus* that can comprise the structural scaffold of *S. aureus* biofilms through self-assembly into functional amyloids (Zaman and Andreasen, 2020). Functional amyloids enhance bacterial resilience to various environmental stresses, augmenting their persistence within the host while concurrently fostering resistance to antimicrobial agents and the immune system (Torrent et al., 2012; Andreasen et al., 2019). Therefore, berberine inhibits the formation of amyloid-like fibers by affecting the aggregation of PSMs, thereby suppressing the formation of the *S. aureus* biofilm and enhancing the bactericidal activity of antibiotics (Chu et al., 2016).

## 11 MD-2

In 2014, we investigated the impact of berberine on *Salmonella Typhimurium* infection. We discovered that berberine could bind to the TLR4/MD-2 receptor complex with higher affinity compared to lipopolysaccharides (LPS) (Chu et al., 2014). MD-2 belongs to the Toll-like receptor (TLR) family and typically forms a complex with the TLR4 protein. This complex is responsible for recognizing and responding to exogenous molecules such as bacterial LPS, leading to the activation of signaling pathways like NF-κB. Consequently, this activation triggers an inflammatory response in immune cells. Excessive activation of TLR4/MD-2 is closely associated with the development of sepsis, endotoxemia, acute lung injury, rheumatoid arthritis, and cardiovascular diseases (Zhang et al., 2022) (Figure 2B).

## 12 BACE1

We investigated the immunological mechanisms and effects of berberine in the treatment of Alzheimer’s disease, revealing that berberine specifically binds to BACE1, one of the key targets in Alzheimer’s disease (Chu et al., 2018). BACE1 is a crucial target in Alzheimer’s disease and holds significance in aging, diabetes, hypertension and cancer (Bao and Shen, 2023). The pathological role of BACE1 in cerebral amyloid angiopathy (CAA) and Alzheimer’s disease has been confirmed in experimental studies. Research has shown that BACE1 expression contributes to the cleavage of amyloid precursor protein (APP) in neurons of APP-overexpressing mice, thereby enhancing the generation of Aβ in neurons (Ihara, 2022). In 2016, Faraco et al. reported that hypertension increases Aβ levels in APP-overexpressing mice by upregulating BACE1 in the brain, although the specific molecular mechanisms, particularly the cell types responsible for the upregulation of BACE1 expression, have not been clarified (Faraco et al., 2016). In 2018, using molecular modeling techniques, we found that berberine was guided into the electronegative binding pocket of BACE1, where the N+ of berberine interacts electrostatically with the crucial anion (Asp80) of BACE1. Additionally, the phenyl group forms a π-π stacking interaction with the Tyr119 active site residue. Furthermore, surface plasmon resonance experiments demonstrated the binding affinity between berberine and BACE1. The equilibrium dissociation constant KD was calculated to be 1.261 μM [(Schumacher et al., 2001; Newberry et al., 2008; Yamasaki et al., 2013; Chandra et al., 2012; Ferraroni et al., 2011; Song et al., 2022; Chen et al., 2022; Zeng et al., 2021)]. As a potential drug molecule, berberine has the ability to bind to BACE1, potentially intervening in Aβ production (Figure 2C).

## 13 Aβ1-42

In addition to BACE1, our study utilizing multi-target drug modeling and surface plasmon resonance experiments identified Aβ 1-42 as a high-affinity target of berberine, suggesting its potential in treating Alzheimer’s disease (Chu et al., 2018). Oligomeric Aβ1-42 is closely associated with neurodegenerative diseases, especially Alzheimer’s disease. It induces oxidative stress and mitochondrial damage in neurons (Thammasart et al., 2023). The aggregation and deposition of Aβ1-42 in the brain are one of the primary mechanisms leading to neuronal damage and cognitive decline (Figure 2C).

## 14 TGFBR1

In 2022, we studied the impact of berberine on lung metastasis in pancreatic cancer and found that berberine can function as a transforming growth factor-beta receptor 1 (TGFBR1) inhibitor, preventing pancreatic cancer cells from breaking through endothelial cells and metastasizing. Through surface plasmon resonance experiments and molecular docking techniques, we determined that the equilibrium dissociation constant (KD) for the binding of berberine to TGFBR1 is 18.0 μM. Berberine interacts with key residues in the active site of TGFBR1, the primary receptor of the TGF-β signaling pathway, including Glu45, Tyr49, Asp81, Tyr82, and His83. It has been demonstrated that when TGF-β molecules bind to the TGFBR2 receptor, TGFBR1 is activated and subsequently transmits the signal into the cell through processes such as phosphorylation, influencing gene expression and cellular behavior. Abnormal activity or mutations in TGFBR1 are associated with various diseases, including cancer, cardiovascular diseases, and immune disorders (Lu et al., 2021; Chen et al., 2020; Xu et al., 2023; Tang et al., 2022). Furthermore, our research showed that berberine inhibits TGFBR1 kinase activity in a dose-dependent manner, with an IC50 of 7.056 μM [(Schumacher et al., 2001; Newberry et al., 2008; Yamasaki et al., 2013; Chandra et al., 2012; Ferraroni et al., 2011; Song et al., 2022; Chen et al., 2022; Zeng et al., 2021)]. This suggests that berberine can serve as an inhibitor in the TGF-β signaling pathway, offering therapeutic potential for cancer, cardiovascular diseases, immune disorders, and more (Figure 2D).

## 15 JAK2

In 2022, we used surface plasmon resonance experiments to confirm the ligand-binding interaction between berberine and JAK2, with a measured KD of 15.83 μM. Additionally, molecular modeling by Song et al. revealed interactions between BBR and specific residues of JAK2, including VAL863, LEU855, LYS857, LEU932, LEU983, GLY993, and ASP994 [(Schumacher et al., 2001; Newberry et al., 2008; Yamasaki et al., 2013; Chandra et al., 2012; Ferraroni et al., 2011; Song et al., 2022; Chen et al., 2022; Zeng et al., 2021)]. Earlier studies have also shown that as a member of the protein tyrosine kinase (PTK) family of JAK proteins, the abnormal activation of JAK2 is closely associated with inflammation, hematopoiesis, malignant tumors, and various age-related diseases (Yang et al., 2021; Fidler et al., 2021; Stevens et al., 2023; Cho et al., 2022). Furthermore, we demonstrated that oral berberine can improve the clinical symptoms of experimental autoimmune myasthenia gravis (EAMG) in rats by reducing the frequency of T helper (TH)1, TH17, and TH1/TH17 cell subsets. We also isolated mononuclear cells (MNCs) from the spleens of EAMG rats and treated them with BBR *in vitro*, finding that the phosphorylation levels of JAK1, JAK2, JAK3, STAT1, and STAT3 were significantly reduced. Similar to JAK2, JAK1 and JAK3 are also likely targets of berberine interaction (Song et al., 2022). In 2023, Huang et al. obtained similar conclusions in a chronic myelogenous leukemia (CML) -like mouse model (Huang et al., 2023) (Figure 2D).

## 16 Conclusion

This review highlights the multiple target actions of berberine in cells and its diverse mechanisms of action. In addition to the 13 berberine targets highlighted in this review, other identified targets include RXRα, ABL1, AKR1B10, and TIGAR (Ruan et al., 2017; Yin et al., 2020; Yang et al., 2024; Qi et al., 2024) (Table 1). Berberine has demonstrated a wide range of pharmacological effects, including anti-inflammatory, anti-tumor, and therapeutic potential in inflammatory diseases, acute and chronic infections, autoimmune disorders, and diabetes. Despite significant progress in understanding these effects, further studies are needed to deepen our understanding of berberine’s specific molecular mechanisms and its broader immunopharmacological properties. While berberine shows promise in various therapeutic areas, several limitations need to be addressed. Current research is largely preclinical, and the translation of these findings into clinical applications remains a challenge, requiring rigorous clinical trials for validation. More research should explore new therapeutic avenues where berberine may offer benefits. In addition, the bioavailability of BBR is rather low after it is absorbed by the gastrointestinal tract which restricts the clinical application. There is an urgent need to enhance the bioavailability of berberine, and further pharmacokinetic studies are warranted.

**TABLE 1:** Identified targets of berberine.

Targets	KD (μM)	IC50 (μM)	Key residues	References
Ftsz	0.02	10.00	Pro 134, Phe 135, Ile 163, Pro 164, Phe 182, Leu 189	Domadia et al. (2008)
QacR	0.72	NA	Trp61, Tyr93, Tyr123, Asn157	Schumacher et al. (2001), Peters et al. (2008)
BmrR	10.30	NA	Phe224, Ile255, Tyr268, Tyr229, Pro224, Pro144	Newberry et al. (2008)
d (CGTACG)2DNA	NA	NA	G6, G8, C5	Ferraroni et al. (2011)
PLA2	5.55	87.00	Gly30, His40, Asp49, Ser23, Cys29, Cys45	Chandra et al. (2011)
RamR	17.90	NA	Site1: Asp152, Met184, Val138, Cys134, Tyr92	Yamasaki et al. (2013), Chu et al. (2018)
			Site2: Asp152, Met184, Val138, Cys134, Tyr92, Leu188, Phe155	
RXRα	30.10	NA	Val242, Glu243, Gln275, Arg316, Arg371	Ruan et al. (2017)
ABL1	0.85	NA	LWEIATYGMSP, NAVVLLYMATQ	Yin et al. (2020)
NEK7	15.60	4.20	Arg121	Zeng et al. (2021)
MET	NA	19.64	Tyr1230, Asp1164	Chen et al. (2022)
AKR1B10	2.07	NA	trp-21, lys-125, gln-303, lys-125, phe-123, trp-220, gly-128	Yang et al. (2024)
TIGAR	4.77	NA	Asn258	Qi et al. (2024)
PSMα2	NA	NA	Phe2, Gly6	Chu et al. (2016)
MD-2	NA	NA	NA	Chu et al. (2014)
BACE1	1.26	62.96	Tyr119, Asp276, Asp80, Val117	Chu et al. (2018)
Aβ1-42	1.49	NA	Val24, Phe19	Chu et al. (2018)
TGFBR1	18.00	7.06	Glu45, Tyr49, Asp81, Tyr82, His83	Tian et al. (2022)
JAK2	15.83	7.40	Val863, Leu855, Lys857, Leu932, Leu983, Gly993, Asp994	Song et al. (2022)

Identified targets of berberine, including their dissociation constant (KD), half-maximal inhibitory concentration (IC50), interacting residues, and associated references. Abbreviations: Aβ1-42, amyloid beta1-42; AKR1B10, aldo-keto reductase 1B10; BACE1, beta-site amyloid precursor protein cleaving enzyme 1; JAK2, janus kinase 2; Ftsz, filamentous temperature-sensitive protein Z; MD-2, myeloid differentiation factor 2; MET, mesenchymal-epithelial transition; NEK7, NIMA-related kinase 7; PLA2, phospholipase A2; PSMα2, phenol-soluble modulins alpha2; RXRα, retinoid X receptor alpha; TGFBR1, transforming growth factor-beta receptor 1; TIGAR, TP53-induced glycolysis and apoptosis regulator.

In conclusion, while berberine holds significant potential, its clinical utility is contingent upon further research and validation, offering both challenges and exciting opportunities for the development of future therapeutic strategies.

## Glossary

AA: arachidonic acid
Aβ1-42: amyloid beta1-42
AKR1B10: aldo-keto reductase 1B10
AMP: adenosine 5′-monophosphate
AMPK: activated protein kinase
APP: amyloid precursor protein
BACE1: Beta-site amyloid precursor protein cleaving enzyme 1
CAA: cerebral amyloid angiopathy
CML: chronic myelogenous leukemia
DTS: Drug-Target Space
EAMG: experimental autoimmune myasthenia gravis
EGFR: epidermal growth factor receptor
Ftsz: filamentous temperature-sensitive protein Z
JAK/STATs: janus kinases/Signal transducer and activator of transcriptions
JAK2: janus kinase 2
LPS: lipopolysaccharides
MAPK: mitogen-activated protein kinases
MBM: multi-target binding motifs
MD-2: myeloid differentiation factor 2
MerR: mercuric -responsive transcriptional regulator
MET: mesenchymal-epithelial transition
MNCs: mononuclear cells
mTOR: mammalian target of rapamycin
NEK7: NIMA-related kinase 7
NF-κB: nuclear factor-κB
NLRP3: nucleotide-binding oligomerization domain-like receptor protein 3
NSCLC: non-small cell lung cancer
PI3K: phosphatidylinositol 3-kinase
PLA2: phospholipase A2
PSMα2: phenol-soluble modulins alpha2
PSMs: phenol-soluble modulins
PTK: protein tyrosine kinase
RXRα: retinoid X receptor alpha
TGF-β: Transforming growth factor β
TGFBR1: transforming growth factor-beta receptor 1
TH: T helper
TIGAR: TP53-induced glycolysis and apoptosis regulator
TLR: Toll-like receptor.
